# [Retracted] Piperine depresses the migration progression via downregulating the Akt/mTOR/MMP-9 signaling pathway in DU145 cells

**DOI:** 10.3892/mmr.2026.13920

**Published:** 2026-05-27

**Authors:** Yuan Zeng, Ying Yang

Mol Med Rep 17: 6363–6370, 2018; DOI: 10.3892/mmr.2018.8653

Following the publication of the above paper, it was drawn to the Editor's attention by a concerned reader that the cellular migration assay data shown in Fig. 5A-C had either appeared previously, or were subsequently to appear, in a handful of other articles in different journals written by different authors at different research institutes, three of which have since been retracted. In addition, an independent analysis of the data in this paper undertaken by the Editorial Office revealed that western blot data featured in Fig. 6A on p. 6368 had also been included in five other papers written by different authors/research groups, the majority of which had already been published or were under consideration for publication when the above article was received at *Molecular Medicine Reports*.

Owing to the fact that the contentious data in the above article had already been published prior to its submission to *Molecular Medicine Reports*, the Editor has decided that this paper should be retracted from the Journal. Upon contacting the authors, the second-listed author, Ying Yang, stated that they were not aware of this submission to the Journal. The Editor apologizes to the readership for any inconvenience caused.

